# HpaB-Dependent Secretion of Type III Effectors in the Plant Pathogens *Ralstonia solanacearum* and *Xanthomonas campestris* pv. *vesicatoria*

**DOI:** 10.1038/s41598-017-04853-9

**Published:** 2017-07-07

**Authors:** Fabien Lonjon, David Lohou, Anne-Claire Cazalé, Daniela Büttner, Barbara Gomes Ribeiro, Claire Péanne, Stéphane Genin, Fabienne Vailleau

**Affiliations:** 1 LIPM, Université de Toulouse, INRA, CNRS, INPT, Castanet-Tolosan, France; 2 Institute of Biology, Genetics Department, Martin Luther University Halle-Wittenberg, D-23 06099 Halle, (Saale) Germany

## Abstract

Plant pathogenic bacteria exerts their pathogenicity through the injection of large repertoires of type III effectors (T3Es) into plant cells, a mechanism controlled in part by type III chaperones (T3Cs). In *Ralstonia solanacearum*, the causal agent of bacterial wilt, little is known about the control of type III secretion at the post-translational level. Here, we provide evidence that the HpaB and HpaD proteins do act as *bona fide R*. *solanacearum* class IB chaperones that associate with several T3Es. Both proteins can dimerize but do not interact with each other. After screening 38 T3Es for direct interactions, we highlighted specific and common interacting partners, thus revealing the first picture of the *R*. *solanacearum* T3C-T3E network. We demonstrated that the function of HpaB is conserved in two phytopathogenic bacteria, *R*. *solanacearum* and *Xanthomonas campestris* pv. *vesicatoria* (*Xcv*). HpaB from *Xcv* is able to functionally complement a *R*. *solanacearum hpaB* mutant for hypersensitive response elicitation on tobacco plants. Likewise, *Xcv* is able to translocate a heterologous T3E from *R*. *solanacearum* in an HpaB-dependent manner. This study underlines the central role of the HpaB class IB chaperone family and its potential contribution to the bacterial plasticity to acquire and deliver new virulence factors.

## Introduction

*Ralstonia solanacearum* species complex (RSSC), the causal agent of bacterial wilt, is divided into four phylotypes (I, II, III and IV), corresponding to different geographical origins of the strains^1^. This devastating plant pathogenic bacterium harbors an unusually large host range as RSSC strains infect more than 250 plant species, including important food crops such as tomato, potato, banana or eggplant, but also model plants such as *Arabidopsis thaliana* and *Medicago truncatula* ^2, 3^. This soilborne pathogen enters into the plant via the roots, colonizes the xylem vessels and then blocks plant water flow driving to wilting symptoms^4^. Beyond the huge arsenal of pathogenicity determinants including exopolysaccharides, plant cell wall degrading enzymes, attachment proteins, and phytohormones, the type III secretion system (T3SS) appears as the main determinant of the bacterial virulence^5^. Indeed, mutants in the genes encoding the T3SS are unable to cause an hypersensitive response (HR) or disease symptoms on host plants^4^.

T3SSs are molecular syringes that allow the injection of bacterial proteins, called type III effectors (T3Es), into the plant cytosol^6^. T3SSs are very well conserved in many plant pathogenic bacteria such as *Xanthomonas* spp., *Pseudomonas syringae* or *Erwinia amylovora* and in animal and human pathogenic bacteria^6^. In phytopathogenic bacteria, the T3SS proteins are encoded by genes located in the *hrp* (hypersensitive response and pathogenicity) gene cluster. The *hrp* clusters are grouped into two families. Hrp1 organization clusters are mostly found in *P*. *syringae* and *E*. *amylovora*, while Hrp2 clusters are found in *R*. *solanacearum* and *X*. *campestris* ^7^.

*R*. *solanacearum* is widely used as a model to study plant-bacteria interactions and has been ranked as the second most important bacterial plant pathogen based on scientific/economic importance^8^. When compared with T3E repertoires of other model phytopathogenic bacteria: *P*. *syringae* (20 to 30 T3Es)^9^, *Xanthomonas* spp. (≈40 T3Es)^10^, and *Erwinia* spp. (10 to 15 T3Es)^11^, the mean repertoire in RSSC strains is larger (48 to 76 T3Es), with a pan-effectome currently comprising 113 T3Es, called Rip for Ralstonia injected proteins^12^. The primary function of T3Es is to hijack plant defenses and to allow bacterial disease development by suppressing plant immunity as well as interfering with plant hormone regulations or plant cellular functions^13^. Alternatively, T3Es can be directly or indirectly recognized by plant resistance proteins, or can activate resistance gene expression, triggering strong plant defenses, leading to the plant resistance^13^. This is the case for the *R*. *solanacearum* effectors RipAA and RipP1 (formerly named AvrA and PopP1), that induce an HR on *Nicotiana* species^14^. The *R*. *solanacearum* GMI1000 strain used in this study possesses 72 Rips^12, 15^. Although many studies have revealed transcriptional control mechanisms of type III secretion^5^, less information is available concerning the post-translational control of T3E secretion.

In plant and animal pathogenic bacteria, two families of proteins have an essential role in type III secretion: the type III chaperones (T3Cs) and the T3S4 (type III secretion substrate specificity switch) proteins. These proteins regulate T3E secretion at post-transcriptional and post-translational levels. They contribute to stabilize and favor the delivery of T3Es into host cells and specifically interact with T3Es, but the importance of these interactions to the secretion process is still not well characterized. It is only hypothesized that these bodyguard proteins may prevent premature interactions of their specific substrates, possibly through binding^16, 17^. It is also described that T3Cs facilitate the directionality and binding of their targeted substrates to components of the secretion apparatus, in particular to the ATPase which energizes the protein translocation through the T3SS^18–20^. T3Cs are generally small and acidic proteins that often interact as homo- or heterodimers with their cognate substrates. These T3Cs either modulate the secretion of one single associated effector (class IA chaperones), several T3Es (class IB chaperones), or the translocon proteins that insert into the host plasma membrane (class II chaperones)^6, 17, 21^. T3S4 proteins interact with the cytoplasmic domains of YscU/HrcU family members (i.e. *Yersinia* YscU, or *Xanthomonas* HrcU family members)^22, 23^ to regulate substrate specificity, in particular allowing the switch between early substrates (including architectural elements of the T3SS) and late substrates (translocon proteins and T3Es)^6, 24–26^. T3S4 proteins interact also with T3Es, but the functional importance of this interaction remains unknown^25^. Up to now, *Xanthomonas campestris* pv. *vesicatoria* 85–10 (called *Xcv*_85-10_ hereafter) is the bacterium for which T3E bodyguards are the best described, especially the HpaB T3C and the T3S4 protein HpaC^18, 25, 27–31^. In *R*. *solanacearum*, secretion helper proteins are called Hpa for Hypersensitive response and pathogenicity associated proteins. In our previous work, we compared the secretomes of the GMI1000 strain with several *hpa* mutants^15^. We showed that the candidate T3Cs studied were playing different roles in T3E secretion. HpaB is required for the secretion of the majority of the T3Es, whereas HpaD is involved in the control of the secretion of few T3Es. We also observed that both proteins are not implicated in T3E stabilization in the bacterial pellets^15^. Up to date, little is known about functional interactions between *R*. *solanacearum* Hpa proteins and T3Es. Our previous research demonstrated that the T3S4 protein HpaP was able to specifically interact with one T3E, RipP1^32^. Here, we investigate the function of two candidate chaperones, HpaB and HpaD, in the post-translational control of *R*. *solanacearum* T3Es. We show that HpaB and HpaD are class IB chaperones interacting with numerous T3Es, and we highlight shared and specific partners. We also studied the role of HpaB in the bacterial virulence. To gain further insights into a potential conserved role of HpaB protein family among the Hrp2 T3SS cluster, the *hpaB* gene from *Xcv*_85-10_ was expressed in the *R*. *solanacearum hpaB* mutant. *Xcv* is the causal agent of bacterial spot disease on pepper and tomato, and has a completely different infectious cycle compared to *R*. *solanacearum*, entering the plant through stomata or wounds to reach the intercellular spaces^33^. Interestingly, we provide evidence of a functional complementation of the *R*. *solanacearum hpaB* mutant, by its *Xcv*_85-10_ counterpart through T3E secretion assays and the ability to induce specific plant responses. On the other hand, we also show that *Xcv*_85-10_ is able to translocate a heterologous *R*. *solanacearum* T3E, highlighting the existence of structurally conserved mechanisms between two phytopathogenic bacteria exhibiting different lifestyles.

## Results

### HpaB and HpaD: two putative type III chaperones conserved among the four phylotypes of *R*. *solanacearum* species complex

**Figure 1:**
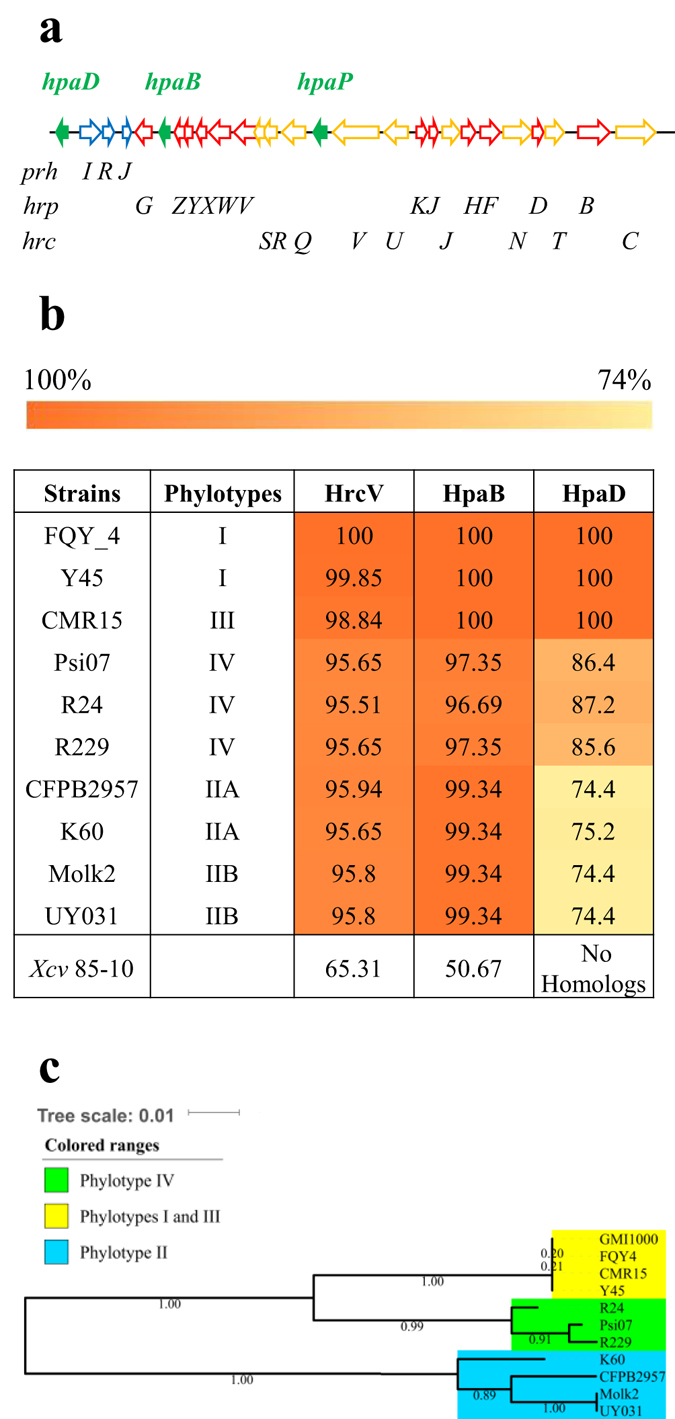
HpaB and HpaD: two putative type III chaperones, encoded by genes located on the *hrp* gene cluster, are conserved among all the four phylotypes of the *R*. *solanacearum* species complex (RSSC). (**a**) Schematic representation of the RSSC *hrp* gene cluster. The arrows represent the different genes. The green, blue, red and yellow color indicates the *hpa*, *prh*, *hrp* and *hrc* genes, respectively. (**b**) Protein identity matrix table of HrcV, HpaB and HpaD from nine RSSC sequenced strains belonging to the four phylotypes compared to the GMI1000 (phylotype I) strain. Matrix cells have been colored from orange to yellow according to the protein conservation (100% orange to 70% yellow). *Xcv*: *Xanthomonas campestris* pv. *vesicatoria*. (**c**) Unrooted phylogenetic tree of HpaD based on sequence similarities using the maximum likelihood method. Ten strains of RSSC were used. Bootstraps were calculated using the approximate likelihood ratio test. The phylogenic tree has been generated using MEGA software.

The *hpaB* gene from *R*. *solanacearum* GMI1000 is localized within the *hrp* gene cluster and the *hpaD* gene is localized on the left border end of the cluster (Fig. 1a). Both genes are conserved among all the sequenced strains of the RSCC. We generated an amino acid conservation matrix table between HpaB, HpaD and HrcV from GMI1000 and other sequenced strains belonging to all the four phylotypes of the RSSC (Fig. 1b). HrcV, a structural core component of the T3SS highly conserved in plant and animal pathogenic bacteria carrying a T3SS, is one of the most conserved protein among the *hrc* (*hrp* conserved)/*hrp* gene cluster (Fig. 1b). The candidate T3C HpaB is highly conserved among the 11 strains, with a higher level of overall sequence identity compared to HrcV proteins (almost 100% identity between strains from phylotypes I, II and III). HpaB is also conserved outside the *Ralstonia* species since HpaB homologs are found in Hrp2 T3SS plant-pathogenic bacteria such as, *Xanthomonas* spp. e.g. *Xcv*_ 85-10_ (50.67% identity), *Xanthomonas oryzae* pv. *oryzae* KACC10331 (64.97% identity), or *Acidovorax citruli* AAC00-1 (74% identity) (Supplementary Fig. S1). The candidate T3C HpaD is also present in the four phylotypes of the RSSC. Although it shows 100% identity between strains from phylotypes I and III, it reaches 85% with strains from phylotype IV, and decreases to around 75% with strains from phylotypes IIA and IIB (Fig. 1b). HpaD homologs were found in other *Ralstonia* species such as *R*. *mannitolilytica* SN83A39 (57.49% identity) or in other uncharacterized *Ralstonia* species (54.03% identity in *Ralstonia* sp. A12). HpaD homologs are found only in close related species such as *Burkholderiaceae* sp. (31.2% identity in *Burkholderiaceae* bacterium 26) (Supplementary Fig. S2). Phylogenetic analysis of HpaD proteins reveals a perfect match with the RSSC phylogeny separating the four phylotypes (Fig. 1c).

### HpaB, but not HpaD, is required for *R*. *solanacearum* pathogenicity on tomato plants

We evaluated the role of the *hpaB* and *hpaD* mutations on *R*. *solanacearum* pathogenicity on tomato plants. First, we checked that the mutants were not affected in growth. For that purpose, we compared the *in vitro* growth kinetics of the *hpaB* and *hpaD* mutants with the wild-type strain in complete medium and in minimal medium supplemented with glutamate by monitoring OD_600nm_ over 25 hours (Supplementary Fig. S3a,b). No significant differences were observed between the three strains in both media. Tomato plants were then root inoculated using the *hpaB* and the *hpaD* mutants and plants survival was monitored daily and compared to the GMI1000 wild-type strain inoculation. The *hpaB* mutant was unable to induce disease on tomato, a phenotype complemented by an ectopic copy of the *hpaB* gene (Supplementary Fig. S3c). On the other hand, HpaD was found to be dispensable for disease establishment on tomato plants, the *hpaD* mutant being able to trigger disease symptoms as the wild-type strain (Supplementary Fig. S3d).

### HpaB and HpaD self-interact but do not interact with each other

**Figure 2:**
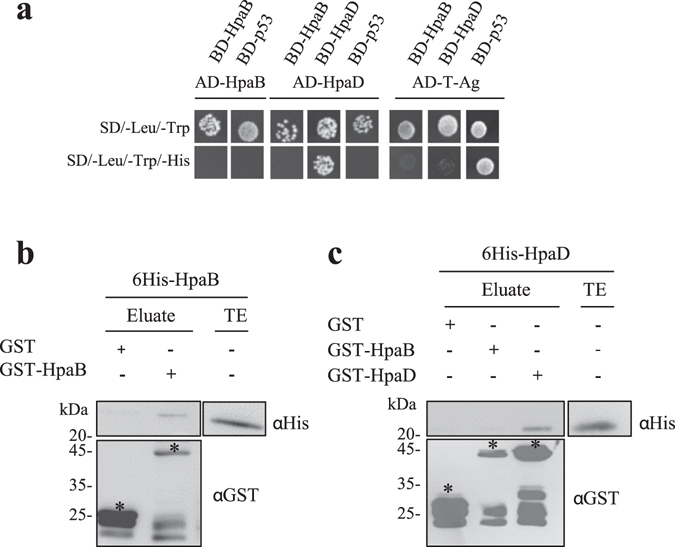
HpaB and HpaD self-interact but do not interact with each other. (**a**) Yeast cells were co-transformed by BD-HpaB or BD-HpaD and AD-HpaB or AD-HpaD. Double transformation and interaction were tested by plating yeasts on synthetic dropout medium lacking leucine and tryptophan (SD/–Leu/–Trp) and synthetic dropout medium lacking leucine, tryptophan and histidine (SD/–Leu/–Trp/–His), respectively. BD-p53 and AD-T-antigen were used as controls, together as a positive control (BD-p53/AD-T-Ag interaction is visualized with a yeast growth on minimal medium lacking histidine) and for tested interactors as negative controls. (**b**) Glutathione S-transferase (GST), GST-HpaB or GST-HpaD were immobilized on glutathione sepharose and incubated with an *E*. *coli* lysate containing 6His-HpaB or 6His-HpaD. Total cell lysates and eluted proteins were analysed using antibodies directed against GST and the 6His epitope. Bands corresponding to GST and GST fusion proteins are marked by asterisks. Lower bands represent degradation products. Two biological replicates were performed for (**a**) and (**b**) with similar results.

We wondered whether candidate T3Cs HpaB and HpaD exhibit known chaperone properties like self-dimerization or if they could act together in the control of type III secretion. Thus, we investigated their ability to self-interact or to interact with each other. Yeast two-hybrid assays were performed using the binding domain (BD) and the activation domain (AD) of the transcription factor GAL4 as fusion partners with HpaB and HpaD. In the case of an interaction between BD- and AD- fusion proteins, yeasts are able to grow on a minimal medium lacking histidine. First, yeasts were co-transformed with the vectors encoding BD-HpaB or BD-HpaD and AD-HpaB or AD-HpaD. BD-p53 and AD-T-antigen were used as controls, both as positive control (BD-p53/AD-T-Ag interaction is visualized with a yeast growth on minimal medium lacking histidine) and as negative control. Indeed, yeasts co-transformed with BD- and AD- (HpaB or HpaD) constructs and one of these controls do not grow on minimal medium lacking histidine (Fig. 2a). This indicated that HpaB and HpaD do not autoactivate in yeast. We observed that HpaD was able to self-interact, while no interaction could be detected between HpaB-HpaB and HpaD-HpaB (Fig. 2a). Then, we looked for potential interactions between these candidate T3Cs using another method. We performed glutathione S-transferase (GST) pull-down assays: GST, GST-HpaB, GST-HpaD fusion proteins were immobilized on glutathione sepharose matrix, and incubated with 6His-HpaB (Fig. 2b) or 6His-HpaD (Fig. 2c). Eluates were analyzed using anti-GST and anti-6His specific antibodies. We were able to detect 6His-HpaB in the presence of GST-HpaB, but not with GST alone (Fig. 2b). Same results were obtained when incubating 6His-HpaD with GST-HpaD (Fig. 2c). 6His-HpaD was not detected when incubated with GST-HpaB (Fig. 2c). All together, these results suggest that both HpaB and HpaD can self-interact whereas HpaB and HpaD do not physically interact.

### HpaB and HpaD each interact with several structurally unrelated T3Es

**Figure 3:**
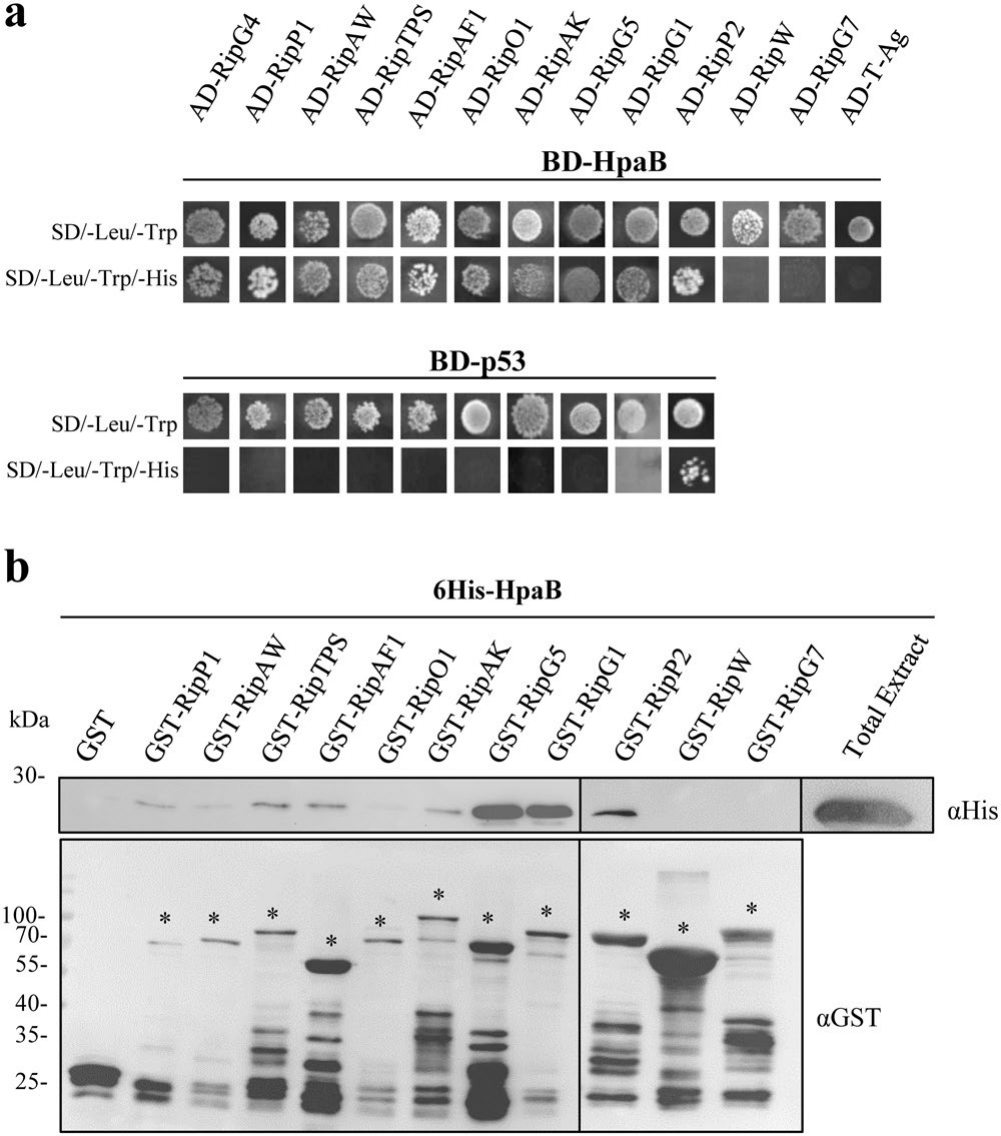
*R*. *solanacearum* HpaB interacts with nine type III effectors. (**a**) Yeast cells were co-transformed by BD-HpaB and 38 AD-T3Es. Double transformation and interactions were tested by plating yeasts on synthetic dropout medium lacking leucine and tryptophan (SD/–Leu/–Trp) and synthetic dropout medium lacking leucine, tryptophan and histidine (SD/–Leu/–Trp/–His), respectively. RipW and RipG7 have the same profile as 26 other T3Es tested. BD-p53 and AD-T-antigen were used as negative controls. Three biological replicates were performed and gave same results. (**b**) Glutathione S-transferase (GST) and GST-T3Es were immobilized on glutathione sepharose and incubated with an *E*. *coli* lysate containing 6His-HpaB. Total cell lysates and eluted proteins were analyzed using antibodies directed against GST and the 6His epitope. Bands corresponding to GST and GST fusion proteins are marked by asterisks; lower bands represent degradation products. For lack of space, samples have been loaded into two gels (black line separation). The experiments have been repeated twice with similar results.

**Figure 4:**
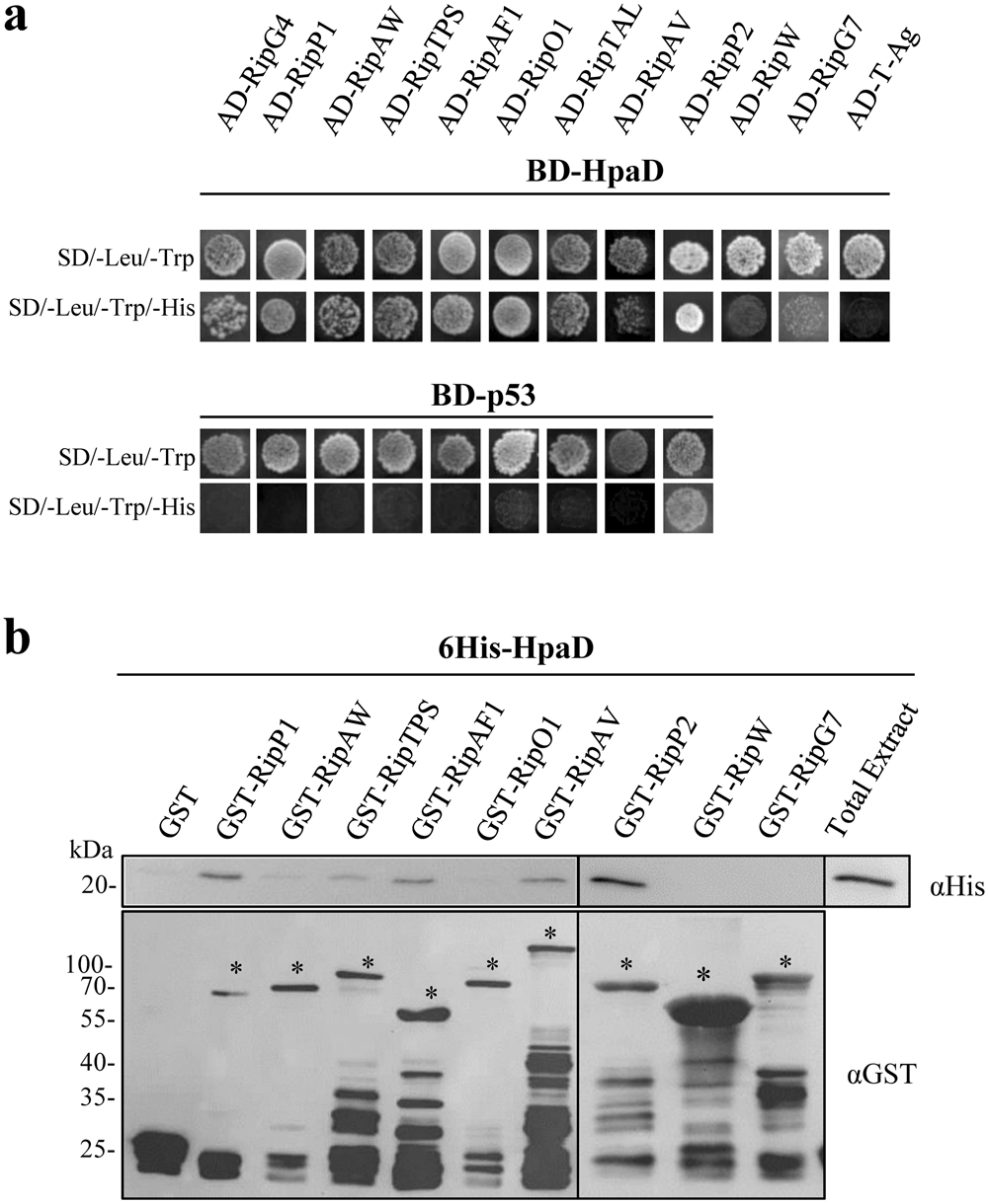
*R*. *solanacearum* HpaD interacts with eight type III effectors. (**a**) Yeast cells were co-transformed by BD-HpaD and 38 AD-T3Es. Double transformation and interactions were tested by plating yeasts on synthetic dropout medium lacking leucine and tryptophan (SD/–Leu/–Trp) and synthetic dropout medium lacking leucine, tryptophan and histidine (SD/–Leu/–Trp/–His), respectively. RipW and RipG7 have the same profile as 27 other T3Es tested. BD-p53 and AD-T-antigen were used as negative controls. Three biological replicates were performed and gave same results. (**b**) Glutathione S-transferase (GST) and GST-T3Es were immobilized on glutathione sepharose and incubated with an *E*. *coli* lysate containing 6His-HpaD. Total cell lysates and eluted proteins were analyzed using antibodies directed against GST and the 6His epitope. Bands corresponding to GST and GST fusion proteins are marked by asterisks; lower bands represent degradation products. For lack of space, samples have been loaded into two gels (black line separation). The experiments have been repeated twice with similar results.

We tested whether HpaB and HpaD act on T3E secretion by direct interactions. Using the yeast two-hybrid method, we screened for HpaB and HpaD interactions with 38 *R*. *solanacearum* T3Es differing in their secretion patterns (from high to intermediate and low levels of secretion, or not detected)^15^. Candidate T3Cs were fused to the binding domain of the GAL4 transcription factor whereas the T3Es were fused to the activation domain. As previously described, we used BD-p53 and AD-T-Ag as control vectors^32^. Because yeasts co-transformed with BD-p53 and AD-RipP2 were able to grow on minimal medium lacking histidine, indicating GAL4 promoter autoactivation in our conditions, we were not able to conclude for HpaB- or HpaD-RipP2 interactions (Figs 3a and 4a). We didn’t detect any autoactivation for the other T3Es tested. Among 37 T3Es-HpaB interactions tested, HpaB was found to interact with nine T3Es, RipG4, RipP1, RipAW, RipTPS, RipAF1, RipO1, RipAK, RipG5 and RipG1, while no interaction was detected for 28 T3Es (Supplementary Table S1). In order to validate these yeast two-hybrid data, we performed GST pull-down experiments with the nine T3Es identified, with RipP2 (as no conclusion could be taken with yeast experiments), and with RipW and RipG7 as negative based on yeast two-hybrid assays. We tested 6His-HpaB in the presence of GST-T3Es. We were not able to express RipG4 as GST-fusion in *E*. *coli*. HpaB was not eluted when incubated with the GST, meaning that HpaB alone does not bind to the GST epitope or to the matrix (Fig. 3b). We were able to identify and confirm a positive interaction with HpaB for RipP1, RipTPS, RipAF1, RipAK, RipG5 and RipG1. A weak signal was visualized for RipAW, suggesting a potential weaker interaction with HpaB. Interestingly, we also revealed a RipP2-HpaB interaction. No signal was observed with RipO1, nor with RipW and RipG7 (Fig. 3b). Same experiments were carried out with the HpaD candidate T3C. First, using yeast two-hybrid assays, among 37 T3Es-HpaD interactions tested, HpaD was found to interact with eight T3Es, RipG4, RipP1, RipAW, RipTPS, RipAF1, RipO1, RipTAL and RipAV, while no interaction was detected for 29 T3Es (Fig. 4a and Supplementary Table S1). We then proceeded as previously to run GST pull-down experiments. Because of toxicity in *E*. *coli* we were not able to express RipTAL as GST-fusion. We were able to identify and confirm a positive interaction with HpaD for RipP1, RipTPS, RipAF1, RipAV, and to highlight an interaction with RipP2. A weak signal was also visualized for RipAW. No signal was observed with RipO1, nor RipW and RipG7 (Fig. 4b).

### The *hpaB* gene from *X*. *campestris* pv. *vesicatoria* can complement the *R*. *solanacearum hpaB* mutant for HR elicitation on tobacco plants

**Figure 5:**
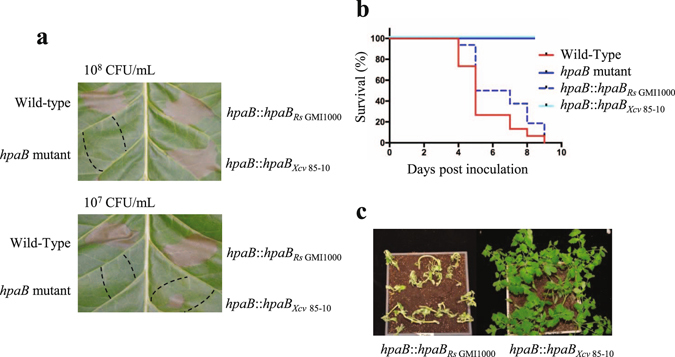
The *hpaB* gene from *X*. *campestris* pv. *vesicatoria* (*Xcv*) 85–10 can complement the *R*. *solanacearum hpaB* mutant to trigger an hypersensitive response (HR) on tobacco plants, but not for disease establishment on tomato plants. (**a**) *N*. *tabacum* leaves were infiltrated with *R*. *solanacearum* bacterial suspensions at 10^8^ CFU/ml (upper part) and 10^7^ CFU/ml (lower part), using the GMI1000 wild-type strain, the *hpaB* mutant (GMI1000 background), the *hpaB* mutant complemented with the *hpaB* gene from *R*. *solanacearum* GMI1000 and the *hpaB* mutant complemented with the *hpaB* gene from *Xcv*_85-10_. Pictures were taken 24 h post infiltration. The dotted lines represent the infiltrated areas. Experiments were repeated three times with similar results. (**b**) Kaplan-Meier survival analysis of 16 tomato plants per *R*. *solanacearum* strain root inoculated. Gehan-Breslow-Wilcoxon test indicates that the wild-type strain curve (red) is significantly different from the *hpaB* mutant curve (blue) (p-value < 0.001), and to the curve showing the *hpaB* mutant complemented with the *Xcv*_85-10_ *hpaB* gene (p-value < 0.001). The wild-type strain curve (red) is not significantly different from the curve showing the *hpaB* mutant complemented with *R*. *solanacearum* GMI1000 *hpaB* gene (dotted cyan) (p-value = 0.0678). Three biological repeats were performed with similar results. (**c**) Representative pictures showing the tomato responses six days after inoculation of the *R*. *solanacearum hpaB* mutant complemented with the *hpaB* genes isolated from *R*. *solanacearum* GMI1000 (left) or *Xcv*_85-10_ (right). Experiments were repeated three times with similar results.

We then conducted a functional characterization of HpaB, as we demonstrated that the protein interacts with numerous effectors, and its defect strongly affects T3E secretion and bacterial pathogenicity. We thus wondered if HpaB could harbor a conserved function among Hrp2 T3SS plant pathogenic bacteria. To study the potential generalist function of HpaB, we generated a strain with a chromosomic insertion of *hpaB*_Xcv 85-10_ into the *R*. *solanacearum hpaB* mutant. The wild-type strain, the *hpaB* mutant and the *hpaB* mutant complemented with the GMI1000 allele (*hpaB*::*hpaB*_*Rs* GMI1000_) or the *Xcv*_ 85-10_ allele (*hpaB*::*hpaB*_*Xcv* 85-10_) were infiltrated into *Nicotiana tabacum* leaves at 10^8^ and 10^7^ CFU/mL. As previously described, the wild-type strain GMI1000 triggers a full HR at 10^7^ and 10^8^ CFU/mL^14^. The *hpaB* mutant does not trigger any HR at both concentrations, and the *hpaB* mutant complemented with *hpaB*_*Rs* GMI1000_ allele restores a full HR at 10^7^ and 10^8^ CFU/mL. Interestingly, complementation with *hpaB*_*Xcv* 85-10_ allele restores a full HR at 10^8^ CFU/mL and a partial HR at 10^7^ CFU/mL (Fig. 5a). The same strains were used to root inoculate tomato plants. While the *hpaB*_*Rs* GMI1000_ allele fully complement the *hpaB* mutation, we observe no disease symptoms with the *hpaB*_*Xcv* 85-10_ allele (Fig. 5b and c).

### Defective secretion of RipP1 and RipP2 in a *R*. *solanacearum hpaB* mutant is partially restored by the *X*. *campestris* pv. *vesicatoria hpaB* gene

**Figure 6:**
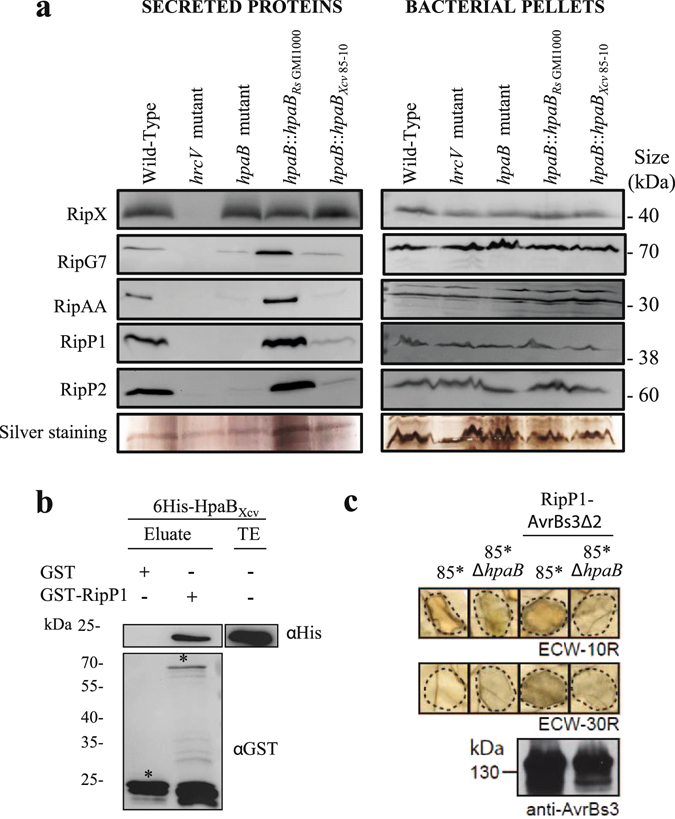
HpaB_*Xcv*_ can functionally control RipP1 secretion/translocation in *R*. *solanacearum* and in *X*. *campestris* pv. *vesicatoria* and is able to bind RipP1. (**a**) Lack of secretion of RipP1 and RipP2 in *R*. *solanacearum hpaB* mutant is partially restored when complementing with the *Xcv*_ 85-10_ *hpaB* gene. *R*. *solanacearum* secretion assays were performed in type III inducing conditions, for the GMI1000 wild-type strain, the *hrcV* mutant, the *hpaB* mutant and the *hpaB* mutant complemented with the *hpaB* gene from *R*. *solanacearum* GMI1000 or the *hpaB* gene from *Xcv*_ 85-10_. Total proteins from bacterial pellets and supernatants were analyzed by Western-blot using specific antibodies or total proteins were stained by silver nitrate. (**b**) HpaB_*Xcv*_ binds *R*. *solanacearum* RipP1 T3E. Glutathione S-transferase (GST) and GST-RipP1 were immobilized on glutathione sepharose and incubated with an *E*. *coli* lysate containing 6His-HpaB_*Xcv*_. Total extract (TE) and eluted proteins were analyzed using antibodies directed against the 6His epitope and GST respectively. Bands corresponding to GST and GST fusion protein are marked by asterisks. (**c**) RipP1 *R*. *solanacearum* T3E is heterogously translocated by *Xcv*, in an HpaB dependent manner. For this experiment, the wild-type strain *Xcv *_85-10_ (85*) and the *hpaB*_*Xcv*_ mutant (85*Δ*hpaB)* both carry hrpG*, a mutated version of the key regulatory gene *hrpG* in the bacterial chromosome (Büttner *et al*., 2004). *Xcv* 85* and 85*Δ*hpaB* strains, and derivatives containing RipP1-AvrBs3Δ2 construct, were infiltrated into leaves of ECW-10R (carrying *Bs1* resistance gene) and ECW-30R (carrying *Bs3* resistance gene) pepper plants. Bacteria were infiltrated at OD_600_ of 0.2 (corresponds to 2 × 10^8^ cfu/ml). Leaves were bleached 2 dpi for better visualization of the HR. Dashed lines indicate the infiltrated areas. For protein analysis, bacteria were grown ON in NYG medium. Equal amounts of cell extracts (adjusted according to OD) were analyzed by SDS-PAGE and immunoblotting, using an AvrBs3-specific antibody. For (**a**), (**b**) and (**c**), two biological replicates were done with similar results.

To study the contribution of a cis-encoded HpaB from *Xcv*_85-10_ on effector secretion in a *R*. *solanacearum hpaB* mutant, we performed secretion assay experiments, growing in T3-inducing conditions the five following strains: the GMI1000 wild-type strain, the *hrcV* mutant (a T3 defective mutant used as a negative control), the *hpaB* mutant and both complemented strains *hpaB*::*hpaB*_*Rs* GMI1000_ and *hpaB*::*hpaB*_*Xcv* 85-10_. Supernatants and cell pellets were harvested and analyzed using specific anti-T3E antibodies. We observed the secretion of five well described T3Es: RipX, RipG7, RipAA, RipP1 and RipP2. Silver staining showed that the same amount of proteins was loaded in each lane. All the five T3Es were detected in same amount in the cell pellets (Fig. 6a). As expected, no T3E was detected in the supernatant of the *hrcV* mutant, indicating no detectable lysis in our samples. As shown in our previous work, specific secretion patterns were observed for the *hpaB* mutant^15^. Indeed, we observed a wild-type secretion of RipX, a partial secretion of RipG7 and no secretion of RipAA, RipP1 and RipP2. T3E secretion was restored in the *hpaB*::*hpaB*_*Rs* GMI1000_ strain. Interestingly, the *hpaB*::*hpaB*_*Xcv* 85-10_ strain was able to partially restore the secretion of RipP1 and RipP2, while the secretion level of RipAA and RipG7 was similar as in the *hpaB* mutant (Fig. 6a). To confirm the physiological relevance for RipP1 secretion in *hpaB*::*hpaB*_*Xcv* 85-10_ complemented strain, we first looked whether HpaB_*Xcv*_ could interact with the *R*. *solanacearum* T3E RipP1 in GST pull-down experiments using HpaB_*Xcv*_ fused to a 6His epitope tag and RipP1 to a GST epitope tag. HpaB_*Xcv*_ was eluted when incubated with the GST-RipP1, but not with GST alone (Fig. 6b), suggesting a possible HpaB_*Xcv*_ - RipP1 interaction.

### *X*. *campestris* pv. *vesicatoria* is able to translocate *R*. *solanacearum* RipP1 effector, in a HpaB-dependent way

We next investigated whether HpaB_*Xcv*_ could function as a chaperone for *R*. *solanacearum* RipP1 T3E, by examining if RipP1 could be translocated by *Xcv*_85-10_. For these experiments, we used the strains 85* and 85*Δ*hpaB* carrying the hrpG* mutation, which confers constitutive activation of the HrpG regulator and, in turn, constitutive expression of the T3SS^34^. We fused RipP1 to the reporter protein AvrBs3Δ2, a derivative of the effector protein AvrBs3 lacking its translocation signal^35^. RipP1-AvrBs3Δ2 fusion construct was then transformed into the strains 85* and 85*Δ*hpaB*. AvrBs3Δ2 contains the effector domain triggering HR in the resistant pepper line ECW-30R carrying the *Bs3* resistance gene^36^ and the fusion protein generated was used to monitor *R*. *solanacearum* RipP1 translocation *in planta*. First, we checked that the 85* strain, but not the 85*Δ*hpaB* mutant, induces the AvrBs1-specific HR when inoculated into the leaves of the AvrBs1-responsive ECW-10R pepper plants^37^. This indicated that RipP1-AvrBs3Δ2 do not interfere with the activity of the T3SS (Fig. 6c). As a second control experiment, we confirmed that after infiltration in ECW30R pepper, 85* strain and 85*Δ*hpaB* mutant do not trigger an HR. Interestingly, after infiltration of the strains carrying RipP1-AvrBs3Δ2 constructs, we observed an HR in ECW-30R pepper plants when delivered by the 85* strain, but not in the 85*Δ*hpaB* mutant (Fig. 6c). Similar amounts of RipP1-AvrBs3Δ2 were detected by immunoblot in total cell extracts for each strain (Fig. 6c), suggesting that the lack of protein translocation by 85*Δ*hpaB* mutant was not due to reduced protein stability. All these data show that *Xcv*_85-10_ is able to translocate a *R*. *solanacearum* T3E, and remarkably, this heterologous translocation is dependent on the HpaB_*Xcv*_ class IB chaperone.

## Discussion

**Figure 7:**
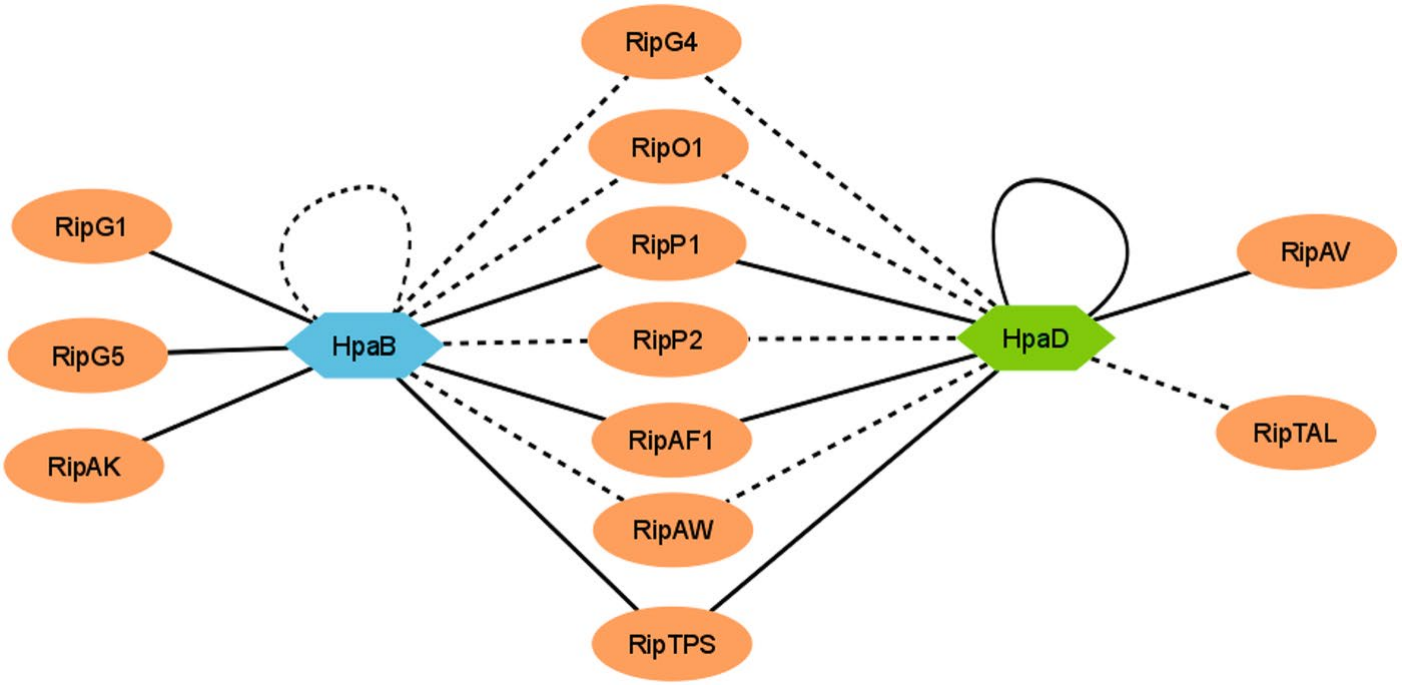
Type III interactome for HpaB and HpaD, two Class IB chaperones of *R*. *solanacearum* exhibit shared and specific T3E partners. This model shows that HpaB and HpaD are able to self-interact, and to interact with numerous T3Es. Solid black lines indicate interactions that have been confirmed by yeast two-hybrid and GST pull-down assays. Dotted black lines indicate interactions that have been identified by one of these two methods. The HpaB-HpaD interacting assays were negative with both methods. The model has been drawn using Cytoscape software.

In *R*. *solanacearum*, HpaB and HpaD were considered as candidate type III chaperones, based on their structural features, their T3-dependent regulation and their requirement for T3E secretion^15^. Both modulate T3E secretion at different levels, but we did not know their roles in terms of functional interactions. In this study, we demonstrated that HpaB and HpaD are *bona fide R*. *solanacearum* class IB chaperones as (i) both are able to form dimeric complexes; and (ii) HpaB and HpaD directly interact with at least ten and nine T3Es, respectively. Moreover, we highlighted seven T3Es as shared interactors while some were demonstrated to interact more specifically, either with HpaB, or HpaD (Fig. 7). Hence, to our knowledge, this study depicts the widest known T3C-T3E interaction network in a phytopathogenic bacterium, validated using two biochemical interaction assays. The homodimerization observed for both studied Hpa proteins is a key feature of numerous T3C-T3E complexes^38^. T3C-T3E interactions were well described in animal and plant pathogenic bacteria, where T3Cs are often clustered with the targeted T3E (Class IA chaperones)^21^. Class IB chaperones have been extensively studied in mammal pathogenic bacteria such as Spa15 of *Shigella flexneri* ^39^, InvB of *Salmonella enterica* ^40, 41^, or CesT of *Escherichia coli* ^42–44^, which each bind to several effectors. In plant pathogenic bacteria, only the HpaB protein, identified specifically in Hrp2 group^7^, has been described as a class IB chaperone in the genus *Xanthomonas* ^6^. HpaB is conserved in all *Xanthomonas* species^45^ and is required for full pathogenicity in *X*. *axonopodis* pv. *glycines* ^45^, *X*. *oryzae* pv. *oryzae* ^46^ and *X*. *campestris* pv. *vesicatoria* ^27^. Several studies in *Xanthomonas* spp. demonstrated that HpaB is important for T3E secretion/translocation^27, 28, 47, 48^. In *Xanthomonas axonopodis* pv. *citri*, a direct interaction was reported with a T3S substrate, HpaA, and with the C-terminal cytosolic domain of HrcV^49^. In *X*. *campestris* pv. *vesicatoria*, HpaB protein interacts with three T3Es, but also with a wide range of non-T3E substrates, such as the inner membrane proteins HrcU and HrcV, the ATPase HrcN, and other T3S control proteins^18, 27, 28, 50–53^. In *R*. *solanacearum*, *hpaB* and *hpaD* are physically linked to the *hrp* gene cluster, suggesting a common evolutionary history. Here, we demonstrated for the first time that both are involved in numerous direct interactions with *R*. *solanacearum* T3Es.

*R*. *solanacearum* HpaB protein appeared to have a more central role. HpaB was previously shown to be required for the efficient secretion and translocation of many *R*. *solanacearum* T3Es^15, 54^. In our previous work^15^, we highlighted three different levels of HpaB-involvement for T3E secretion: (1) T3Es secreted in an HpaB-independent way; (2) T3Es for which secretion is partially dependent on HpaB; and (3) T3Es secreted in a strict HpaB dependency. For T3Es belonging to the two first classes (for RipW, RipAM and for RipG7, respectively), no interaction was observed. Otherwise, all the T3Es identified as HpaB-interactors are strictly dependent on HpaB for their secretion (RipG5, RipP1, RipP2, RipAF1 and RipAK). Hence, these data reinforce the HpaB-independent T3S pattern for the first classes of substrates. For the other T3Es, whose secretion is HpaB-dependent, numerous direct interactions have been highlighted. Among the large number of T3Es requiring HpaB for their secretion^15^, at least 28 T3Es were not identified as direct HpaB-interactors in this study. Because it is known that a major limiting factor of yeast two-hybrid is the false negative rate of the assays^55^, we cannot exclude the occurrence of such false negative interactions in our experiments and that consequently a large part of these 28 T3Es may be indirectly controlled by HpaB. We can hypothesize that these 28 T3Es may be indirectly controlled by HpaB for their secretion, involving other chaperones or uncharacterized co-factors. Moreover we cannot exclude that HpaB controls T3E secretion through interactions with structural components of the T3SS. Otherwise, while HpaB and HpaD share several interacting partners, we could not identify a direct HpaB-HpaD interaction, using two different techniques. Other studies described that two T3Cs could interact with the same translocon protein^56–58^. To our knowledge, we describe here the first example of two T3Cs interacting with the same T3Es (seven common interactors identified). However, their implication in *R*. *solanacearum* pathogenicity is different. We demonstrated that HpaB is strictly required for disease establishment and to trigger an HR on tobacco plants. This is explained by the defect of the secretion of numerous T3Es in the *hpaB* mutant^15^. Differently, even if binding to at least nine T3Es, we previously observed for the *hpaD* mutant a secretion pattern close to the wild-type strain, and we were not able to see an implication of HpaD on bacterial pathogenicity of *R*. *solanacearum* GMI1000^15^. The *hpaD* gene seems to be restricted to *Ralstonia* species or to very close related species. In the RSSC, we saw that HpaD sequences diverged into two distinct phylogenetic groups. We can hypothezise that HpaD could have a more important role in other strains belonging to phylotype II or IV or could be important for pathogenicity on specific hosts. Its exact function remains to be elucidated.

The *R*. *solanacearum* HpaB protein sequence is highly conserved among the RSSC (97 to 100% identity), but HpaB_*Rs* GMI1000_ shares only 50.67% identity with HpaB_*Xcv* 85-10_. This raised the question of the functional conservation of HpaB class IB chaperone among Hrp2 plant pathogenic bacteria. We therefore investigated if HpaB proteins from *R*. *solanacearum* and *Xcv*, two phytopathogenic bacteria with different lifestyles, different modes in invasions, exhibiting different repertoires of T3Es and different host plants, were functionally interchangeable. Interestingly, trans-complementation for T3S using T3Es^59^, T3S regulators^60^, or T3Cs^61^ from other organisms was previously studied, mainly showing heterologous secretion or heterologous interactions. In this study, we functionally complement the *R*. *solanacearum hpaB* mutant to trigger an HR on tobacco plants using HpaB_*Xcv* 85-10_. We also show that secretion of RipP1 and RipP2 can be directed by HpaB_*Xcv* 85-10_ in *R*. *solanacearum*. This may explain the tobacco HR phenotype observed, consistent with a previous study showing that RipP1 secretion was required to trigger a full HR on tobacco plants at high concentrations^14^. We also observe that some common T3S features are conserved between the two phytopathogenic bacteria, as *R*. *solanacearum* RipP1 binds to HpaB_*Xcv* 85-10_ class IB chaperone, and *Xcv* is able to efficiently translocate a heterologous *R*. *solanacearum* T3E, in a HpaB_*Xcv* 85-10_ dependent manner. However, HpaB_*Rs* GMI1000_ and HpaB_*Xcv* 85-10_ are not fully interchangeable. Indeed, ectopic expression of *hpaB*_*Xcv* 85-10_ in *R*. *solanacearum hpaB* mutant leads to a partial complementation. *hpaB*_*Xcv* 85-10_ restores the HR phenotypes on tobacco, but not disease establishment on tomato. Pathogenicity of *R*. *solanacearum* on tomato is multifactorial, depending on numerous and functionally redundant T3Es^62^. This probably explains why the disease phenotype cannot be complemented whereas RipP1 alone is sufficient for elicitation of the HR^14^.

Another interesting result in our study is the fact that a *R*. *solanacearum* T3E can be translocated by *Xcv*_85-10_ into pepper plant cells. This observation suggests that such class IB chaperones could play a role in the rapid adaptation of the pathogen by ensuring type III-dependent translocation of recently acquired T3Es (through horizontal gene transfer for example)^12, 63^. *R*. *solanacearum* is a remarkable plant pathogen as the RSSC comprises meta-repertoires of more than 100 T3E families, and 36 out of these T3E families share homologies with T3Es identified in the plant pathogenic bacteria *Pseudomonas syringae*, *Xanthomonas* and *Acidovorax* spp.^62^. Hence, the evolution of bacterial pathogenicity is clearly dynamic and loss or gain of T3Es can then lead to host range shifts. This abundance of virulence factors requires helper proteins to avoid uncontrolled delivery and a fine orchestration of T3E delivery presumably exists^64^. T3SSs are regulated by common mechanisms in different bacterial species^6^, and we now know that T3Cs, like HpaB class IB chaperones, are essential in this fine control and may significantly contribute to the bacterial plasticity to acquire or deliver new virulence factors.

## Methods

### Bacterial strains and media

The bacterial strains used in this work are listed in Supplementary Table S2. *E*. *coli* strains were grown at 37 °C in Luria–Bertani medium. *R*. *solanacearum* strains were grown in complete BG medium or in MP minimal medium as previously described^65^. The MP minimal medium was adjusted to pH 6.5 with KOH. When needed, antibiotics were added at the following final concentrations (mg/L): kanamycin (50), spectinomycin (40), gentamycin (10), tetracycline (10) for *R*. *solanacearum* and kanamycin (25), gentamycin (10), tetracycline (10), ampicillin (50), chloramphenicol (25) for *E*. *coli*.

### *R*. *solanacearum in vitr*o growth

The growth curves of the wild-type strain, the *hpaB* mutant and the *hpaD* mutant were measured in two culture media: complete BG medium and MP minimal medium supplemented with glutamate at a 20 mM final concentration. Overnight cultures were used to inoculate four replicates of 200 µl of fresh medium with an initial OD_600nm_ at 0.1. Bacterial growth was performed in 96-well plates and monitored every 5 min using the FLUOstar Omega microplates reader (BMG Labtech, France). Experiments were repeated twice.

### Phylogenetic analysis

The evolutionary history was inferred by using the Maximum Likelihood method based on the Whelan and Goldman model^66^. The tree with the highest log likelihood (-653.1786) is shown. The percentage of trees in which the associated taxa clustered together is shown next to the branches. Initial tree(s) for the heuristic search were obtained automatically by applying Neighbor-Join and BioNJ algorithms to a matrix of pairwise distances estimated using a Jones-Taylor-Thornton model^67^, and then selecting the topology with superior log likelihood value. The tree is drawn to scale, with branch lengths measured in the number of substitutions per site. All positions containing gaps and missing data were eliminated. There were a total of 125 positions in the final dataset. Evolutionary analyses were conducted in MEGA6^68^. The tree were manipulated and annotated using Interactive Tree Of Life tool (http://itol.embl.de/)^69^. The analysis involved 11 amino acid sequences belonging to the four phylotypes selected strains: GMI1000^70^, Y45^71^ and FQY_4^72^ from the phylotype I, CFBP2957^73^ and K60^74^ from phylotype IIA, Molk2^73^ and UY031^75^ from phylotype IIB, CMR15^73^ from phylotype III and Psi07^73^, R229^76^ and R24^76^ from phylotype IV. To generate protein matrix identity, sequences were aligned using MUSCLE^77^. Then proteins identities were calculated using SIAS tool (http://imed.med.ucm.es/Tools/sias.html).

### Cloning

The plasmids used in this study and listed in Supplementary Table S2, were constructed by Gateway^TM^ technology (Invitrogen, Carlsbad, CA, USA). Plasmids in pENTR SD/D TOPO background were generated following manufacturer’s instructions using specific primers listed in Supplementary Table S3. Genes cloned in the pDONR207 plasmid were amplified in two steps. The first PCR was performed using the specific primers indicated in Supplementary Table S3. The second PCR was performed using 1 µL of the first PCR as matrix and oNP291-oNP292 universal primers. PCR products were cloned into pDONR207 using Gateway^TM^ BP reaction following manufacturer’s conditions. Final plasmids for yeast two-hybrid, GST pull-down, and stable integration in a large non-coding chromosomic region downstream of the *glmS* gene in *R*. *solanacearum* ^78^ were generated using Gateway^TM^ LR reaction.

Vectors for translocation assays using *Xcv* 85* strain and 85*Δ*hpaB* mutant were generated by Golden Gate cloning. First, RipP1 was amplified with compatible *BsaI* site and ligated into pGEM-T vector (Promega, Madison, WI, USA) to generate pFL92. Then, pFL92 was mixed with pBR356, *BsaI* restriction enzyme and T4 DNA ligase in a thermomixer using the following conditions: 37 °C for 1 hour, 50 °C for 10 min, 65 °C for 20 min to generate pFL98.

### Yeast two-hybrid assays

Yeast two-hybrid experiments were performed as previously described^32^. *Saccharomyces cerevisiae* strain AH109 was cotransformed, using the LiAc methods^79^, with BD and AD fusions and plated on minimal synthetic-dropout (SD) medium (lacking leucine and tryptophan). Double transformants were tested for interaction by platting successive dilutions on minimal SD medium lacking leucine, tryptophan and histidine. Each interaction test was performed on four different colonies. Two to three biological repetitions were made for each interaction tested.

### GST pull-down assays

GST fusion and 6His fusion proteins were synthesized in *E*. *coli* BL21(DE3). Bacterial cells from 50 mL cultures were resuspended in Phosphate Buffer Salt (PBS) and lysated with a French press. Cellular debris were eliminated by centrifugation. GST and GST fusion proteins were immobilized on a glutathione sepharose matrix, preliminarily washed with PBS, for 30 minutes. Unbound proteins were washed with PBS, and the glutathione sepharose matrix was incubated with 500 µl *E*. *coli* cell lysates containing the 6His fusion proteins for 2 hours. Unbound proteins were washed with PBS. Proteins were eluted with 10 mM reduced glutathione. Eluted proteins and protein lysates were analyzed by SDS-PAGE and immunoblotting using antibodies specific for the 6His epitope and GST (Roche Applied Science, Mannheim, Germany). Experiments were repeated at least twice.

### Pathogenicity assays

16 plants of *Solanum lycopersicum* cv. Marmande VR, from 3-4-week-old plants, were soaked with 500 mL of a bacterial suspension at 5.10^7^ bacteria/mL. Disease development was monitored every day, and plants with at least 50% of wilting were considered as dead for the statistical survival analysis. To compare the disease development of two given strains, we used the Kaplan–Meier survival analysis with the Gehan-Breslow-Wilcoxon test^80^. A p-value smaller than 0.05 was considered significant, indicating that the Ho hypothesis of similarity of the survival experience of the tested strains can be rejected. Statistical analyses were done with Prism version 5.00 (GraphPad Software, San Diego, CA, USA). Each experiment was repeated at least three times.

### Secretion assays

The pAM5 plasmid was introduced by electroporation into the wild-type strain, the *hrcV* mutant, the *hpaB* mutant and the *hpaB* mutant complemented versions: *hpaB*::*hpaB*_*Rs* GMI1000_ and *hpaB*::*hpaB*_*Xcv* 85-10_, to obtain a higher transcriptional activity of T3SS-regulated genes. Secretion assays and Western blot analysis were performed as previously described^15^. Antibodies used for Western blotting were RipG7^32^, RipP1^81^, RipP2 (courtesy of L. Deslandes, LIPM, INRA/CNRS, Castanet Tolosan, France), RipX^82^ and RipAA^14^. Goat anti-rabbit antibody conjugated with horseradish peroxidase was used as secondary antibody (Santa Cruz Biotechnology, Santa Cruz, CA, U.S.A.). For silver nitrate staining, samples were loaded onto a SDS-PAGE bisacrylamid gel. After migration, proteins were stained using Silver Stain Plus Kit (Bio-Rad, USA) following manufacturer conditions. Two biologically independent experiments were performed.

### *In vivo* translocation assays and immunoblot analyses

Translocation assays were performed mostly as previously described^83^. *Xcv* strains (85* and 85*Δ*hpaB* mutant) were infiltrated into leaves of 4-week-old ECW-10R pepper plants (carrying *Bs1* resistance gene), and ECW-30R pepper plants (carrying *Bs3* resistance gene) at a concentration of 4.10^8^ CFU/ml. Pepper plants were then incubated for 16 h light at 28 °C/8 h darkness at 22 °C, and 65% humidity. The appearance of HR was evaluated 2 days post infiltration. Leaves were destained in 70% ethanol. For verification of RipP1-AvrBs3Δ2 equal amounts of bacterial total cell extracts were analyzed by SDS-PAGE and immunoblotting using antibodies specific for AvrBs3^84^. Horseradish peroxidase labeled anti-rabbit antibodies (GE Healthcare, Buckinghamshire, UK) was used as secondary antibodies. Experiments were performed twice.

## Electronic supplementary material

Supplementary information
