# Supplementary material for: HpaB-Dependent Secretion of Type III Effectors in the Plant Pathogens Ralstonia solanacearum and Xanthomonas campestris pv. vesicatoria
Source: Sci Rep. 2017 Jul 7;7:4879. doi: 10.1038/s41598-017-04853-9 (PMC5501821; doi:10.1038/s41598-017-04853-9)
Supplement: Supplementary file 1 — Supplementary information [file 41598_2017_4853_MOESM1_ESM.pdf]

Supplementary informations

HpaB-Dependent Secretion of Type III Effectors in the Plant Pathogens *Ralstonia solanacearum* and *Xanthomonas campestris* pv. *vesicatoria*

Fabien Lonjon[1], David Lohou[1], Anne-Claire Cazalé[1], Daniela Büttner[2], Barbara Gomes Ribeiro[1], Claire Péanne[1], Stéphane Genin[1], Fabienne Vailleau[1,]*

[1]LIPM, Université de Toulouse, INRA, CNRS, INPT, Castanet-Tolosan, France

[2]Institute of Biology, Genetics Department, Martin Luther University Halle-Wittenberg, D-23 06099 Halle (Saale), Germany

*fabienne.vailleau@inra.fr

Supplementary Table S1: Results of the interactions between *Ralstonia solanacearum* type III effectors and HpaB and HpaD proteins after yeast two-hybrid assays.

***R. solanacearum* type III effector**	***R. solanacearum* corresponding *gene***	**Interaction with HpaB in yeast**	**Interaction with HpaD in yeast**
RipA1	*RSc2139*	-	-
RipA2	*RSp0099*	-	-
RipA3	*RSp0846*	-	-
RipA4	*RSp0847*	-	-
RipA5	*RSp1024*	-	-
RipB	*RSc0245*	-	-
RipG1	*RSp0914*	+	-
RipG3	*RSp0028*	-	-
RipG4	*RSc1800*	+	+
RipG5	*RSc1801*	+	-
RipG6	*RSc1356*	-	-
RipG7	*RSc1357*	-	-
RipH1	*RSc1386*	-	-
RipH2	*RSp0215*	-	-
RipH3	*RSp0160*	-	-
RipI	*RSc0041*	-	-
RipO1	*RSp0323*	+	+
RipP1	*RSc0826*	+	+
RipP2	*RSc0868*	Autoactivation	Autoactivation
RipQ	*RSp1277*	-	-
RipR	*RSp1281*	-	-
RipU	*RSp1212*	-	-
RipV1	*RSc1349*	-	-
RipW	*RSc2775*	-	-
RipZ	*RSp1031*	-	-
RipAD	*RSp1601*	-	-
RipAF1	*RSp0822*	+	+
RipAG	*RSc0824*	-	-
RipAJ	*RSc2101*	-	-
RipAK	*RSc2359*	+	-
RipAM	*RSc3272*	-	-
RipAN	*RSp0845*	-	-
RipAV	*RSp0732*	-	+
RipAW	*RSp1475*	+	+
RipAY	*RSp1022*	-	-
RipAZ1	*RSp1582*	-	-
RipTAL	*RSc1815*	-	+
RipTPS	*RSp0731*	+	+

Supplementary Table S2: Strains and plasmids used in this study.

	Characteristics	Source
***R. solanacearum* strains**		
GMI1000	Wild-type strain	1
GMI1694	GMI1000 *hrcV*::Ω mutant, Sp^r	2
GRS266	GMI1000 *hpaD*::Ω mutant, Sp^r	2
GRS474	GMI1000 *hpaB*::Ω mutant, Sp^r	3
***X. campestris* pv. *vesicatoria* strains**		
85-10	wild type strain; Rif^r	4
85*	85-10 derivative containing the *hrpG** mutation, Rif^r	5
85*Δ*hpaB*	*hpaB* deletion mutant (deleted in codons 13 to 149) of strain 85*, Rif^r	6
Yeast strain		
AH109	Matchmaker™ yeast strain	BD Biosciences, Palo Alto, CA, USA
E.coli strain		
BL21(DE3)	F^- *ompT gal dcm lon hsdSB*(r_B^- m_B^-)	Invitrogen, Carlsbad, CA, USA
Rosetta	*F- ompT hsdSB(rB- mB-) gal dcm (DE3) pRARE (CamR)*	Invitrogen, Carlsbad, CA, USA
**Plasmids used for cloning**		
pACC61	pDONR207 derivative carrying *ripAZ1*, Gm^r	This study
pACC73	pDONR207 derivative carrying *ripAF1*, Gm^r	This study
pACC75	pDONR207 derivative carrying *ripO1*, Gm^r	This study
pACC82	pDONR207 derivative carrying *ripZ*, Gm^r	This study
pACC93	pDONR207 derivative carrying *ripAM*, Gm^r	This study
pACC98	pDONR207 derivative carrying *ripH2*, Gm^r	This study
pACC113	pDONR207 derivative carrying *ripAK*, Gm^r	This study
pACC221	pDONR207 derivative carrying *ripAN*, Gm^r	This study

pACC271	pDONR207 derivative carrying *ripAW*, Gm^r	This study
pACC276	pDONR207 derivative carrying *ripQ*, Gm^r	This study
pACC385	pDONR207 derivative carrying *ripAY*, Gm^r	This study
pACC386	pDONR207 derivative carrying *ripH1*, Gm^r	This study
pACC426	pDONR207 derivative carrying *ripR*, Gm^r	This study
pACC429	pDONR207 derivative carrying *ripW*, Gm^r	This study
pACC532	pENTR/D-TOPO derivative carrying *ripTAL*, Km^r	Thomas Lahaye, Universität Tübingen
pACC542	pDONR207 derivative carrying *ripAG*, Gm^r	This study
pACC546	pDONR207 derivative carrying *ripV1*, Gm^r	This study
pACC588	pDONR207 derivative carrying *ripH3*, Gm^r	This study
pACC648	pENTR/SD/D-TOPO derivative carrying *ripAD*, Km^r	This study
pACC652	pDONR207 derivative carrying *ripAJ*, Gm^r	This study
pBR356	Vector carrying *avrBs3Δ2* downstream of the lac promoter and the lacZ fragment flanked by *BsaI* sites	7
pDONR207	Gateway™ entry vector, Gm^r Cm^r	Invitrogen, Carlsbad, CA, USA
pENTR/SD/D-TOPO	Gateway™ entry vector, Shine–Dalgarno sequence, Km^r	Invitrogen, Carlsbad, CA, USA
pFL47	pENTR/SD/D-TOPO derivative carrying $hpaB_{Xcv85\text{-}10}$, Km^r	This study
pFL92	pGEM-T derivative carrying *ripP1* flanked by *BsaI* site	This study
pGAD	Matchmaker™ pGADT7 yeast expression vector made Gateway™ compatible, N-terminal AD fusion, Ap^r, Cm^r	8
pGBG	Matchmaker™ pGADT7 yeast expression vector made Gateway™ compatible, N-terminal BD fusion, Ap^r, Cm^r	8
pGEX	Gateway™ expression vector, N-terminal GST fusion, Ap^r	Invitrogen, Carlsbad, CA, USA
pGEM-T	TA cloning vector, lacZα,f1 ori, Amp^r	Promega, Madison, WI, USA
pLBy1	pDONR207 derivative carrying *hpaD*, Gm^r	This study
pLBy3	pDONR207 derivative carrying *ripI*, Gm^r	This study

pLBy5	pDONR207 derivative carrying *hpaB*, Gm^r	3
pLby7	pDONR207 derivative carrying *ripU*, Gm^r	This study
pLBy13	pDONR207 derivative carrying *ripAV*, Gm^r	This study
pMP12	pDONR207 derivative carrying *ripTPS*, Gm^r	9
pMS130	pENTR/SD/D-TOPO derivative carrying *ripA2*, Km^r	10
pMS132	pDONR207 derivative carrying *ripA5*, Gm^r	10
pMS456	pDONR207 derivative carrying *ripA3*, Gm^r	10
pMS457	pDONR207 derivative carrying *ripA1*, Gm^r	10
pMS459	pENTR/SD/D-TOPO derivative carrying *ripA4*, Km^r	10
pNP200	pDONR207 derivative carrying *ripG7*, Gm^r	11
pNP329	pRCG derivative with the *ripG7* promoter followed by a Gateway™ destination cassette and a triple HA epitope tag, Gm^r Km^r	8
pPR122	pDONR207 derivative carrying *ripG6*, Gm^r	11
pPR126	pDONR207 derivative carrying *ripG4*, Gm^r	11
pPR134	pDONR207 derivative carrying *ripG5*, Gm^r	11
pPR156	pDONR207 derivative carrying *ripG3*, Gm^r	11
pPR181	pDONR207 derivative carrying *ripG1*, Gm^r	11
pSC192	pENTR/SD/D-TOPO derivative carrying ripB, Km^r	This study
pTH19	Gateway™ expression vector, N-terminal 6His tag, Ap^r	Invitrogen, Carlsbad, CA, USA
**Plasmids for yeast two hybrid**		
pDL6	pGBG derivative carrying *hpaB*, Km^r	This study
pDL7	pGBG derivative carrying *hpaD*, Km^r	This study
pDL9	pGAD derivative carrying *ripI*, Amp^r	This study
pDL10	pGAD derivative carrying *ripU*, Amp^r	This study
pDL12	pGAD derivative carrying *ripAV*, Amp^r	This study

pDL13	pGAD derivative carrying *ripAG*, Amp^r	This study
pDL14	pGAD derivative carrying *ripAD*, Amp^r	This study
pDL15	pGAD derivative carrying *ripAZ1*, Amp^r	This study
pDL16	pGAD derivative carrying *ripAJ*, Amp^r	This study
pDL17	pGAD derivative carrying *ripAF1*, Amp^r	This study
pDL18	pGAD derivative carrying *ripO1*, Amp^r	This study
pDL19	pGAD derivative carrying *ripZ*, Amp^r	This study
pDL20	pGAD derivative carrying *ripAM*, Amp^r	This study
pDL21	pGAD derivative carrying *ripH2*, Amp^r	This study
pDL22	pGAD derivative carrying *ripAK*, Amp^r	This study
pDL23	pGAD derivative carrying *ripAN*, Amp^r	This study
pDL24	pGAD derivative carrying *ripAW*, Amp^r	This study
pDL25	pGAD derivative carrying *ripQ*, Amp^r	This study
pDL26	pGAD derivative carrying *ripV1*, Amp^r	This study
pDL27	pGAD derivative carrying *ripAY*, Amp^r	This study
pDL28	pGAD derivative carrying *ripH1*, Amp^r	This study
pDL29	pGAD derivative carrying *ripR*, Amp^r	This study
pDL30	pGAD derivative carrying *ripW*, Amp^r	This study
pDL33	pGAD derivative carrying *ripA2*, Amp^r	This study
pDL34	pGAD derivative carrying *ripA4*, Amp^r	This study
pDL35	pGAD derivative carrying *ripA3*, Amp^r	This study
pDL36	pGAD derivative carrying *ripA5*, Amp^r	This study
pDL37	pGAD derivative carrying *ripA1*, Amp^r	This study
pDL38	pGAD derivative carrying *ripTPS*, Amp^r	This study
pDL40	pGAD derivative carrying *ripG4*, Amp^r	This study

pDL41	pGAD derivative carrying *ripG5*, Amp^r	This study
pDL42	pGAD derivative carrying *ripG6*, Amp^r	This study
pDL43	pGAD derivative carrying *ripG7*, Amp^r	This study
pDL44	pGAD derivative carrying *ripB*, Amp^r	This study
pDL45	pGAD derivative carrying *ripG1*, Amp^r	This study
pDL46	pGAD derivative carrying *ripG3*, Amp^r	This study
pDL47	pGAD derivative carrying *ripH3*, Amp^r	This study
pDL48	pGAD derivative carrying *ripTAL*, Amp^r	This study
pDL50	pGAD derivative carrying *hpaB*, Amp^r	This study
pDL51	pGAD derivative carrying *hpaD*, Amp^r	This study
pDL53	pGAD derivative carrying *ripP1*, Amp^r	This study
pDL54	pGAD derivative carrying *ripP2*, Amp^r	This study
pAD-T-Ag	AD-t-antigen fusion	BD Biosciences, Palo Alto, CA, USA
pBD-p53	BD-p53 fusion, Km^r	BD Biosciences, Palo Alto, CA, USA
**Plasmids for GST-Pulldown**		
pDL175	pGEX derivative carrying *hpaD*, Amp^r	This study
pDL188	pGEX derivative carrying KQGS*, Apr	8
pDL193	pTH19 derivative carrying *hpaD*, Amp^r	This study
pFL37	pTH19 derivative carrying *hpaB*, Amp^r	This study
pFL67	pGEX derivative carrying *ripAF1*, Amp^r	This study
pFL68	pGEX derivative carrying *ripAK*, Amp^r	This study
pFL69	pGEX derivative carrying *ripG5*, Amp^r	This study
pFL70	pGEX derivative carrying *ripG1*, Amp^r	This study
pFL71	pGEX derivative carrying *ripG7*, Amp^r	This study
pFL72	pGEX derivative carrying *ripTPS*, Amp^r	This study

pFL75	pGEX derivative carrying *ripAW*, Amp^r	This study
pFL76	pGEX derivative carrying *ripO1*, Amp^r	This study
pFL77	pGEX derivative carrying *ripW*, Amp^r	This study
pFL83	pGEX derivative carrying *ripP1*, Amp^r	This study
pFL84	pGEX derivative carrying *hpaB*, Amp^r	This study
pFL116	pTH19 derivative carrying $hpaB_{Xcv}$, Amp^r	This study
pGEX-RipP1	pGEX derivative carrying *ripP1*, Amp^r	Laurent Deslandes, LIPM
pGEX-RipP2	pGEX derivative carrying *ripP2*, Amp^r	12
**Plasmids transformed in *R. solanacearum***		
pAM5	pLAFR3 carrying 2-kb fragment containing *hrpB*,Tc^r	13
pEG11	pNP329 derivative carrying $hpaB_{\mathrm{GMI1000}}$, Km^r, Gm^r	3
pFL49	pNP329 derivative carrying $hpaB_{Xcv\ 85\text{-}10}$, Km^r, Gm^r	This study
**Plasmids transformed in *X. campestris* pv. *vesicatoria***		
pFL98	pBR356 derivative carrying *ripP1*, Gm^r	This study

Supplementary Table S3: List of primers used in this study.

	Gene	Recipient Plasmid	Plasmid ID	Primers	Primer Sequences
RipAZ1	*RSp1582*	pDONR207	pACC61	Oacc31<br>Oacc32	GGAGATAGAACCATGCCCCCATCGATCCG<br>CAAGAAAGCTGGGTCTCMCACCGGGAATGCTTTCC
RipAF1	*RSp0822*	pDONR207	pACC73	Oacc19<br>Oacc20	GGAGATAGAACCATGGGTTTGCCACGGATC<br>CAAGAAAGCTGGGTCTCMTCGCGTTGACGTGGACG
RipO1	*RSp0323*	pDONR207	pACC75	Oacc17<br>Oacc18	GGAGATAGAACCATGCCAAAAATCCCAAAAAACC<br>CAAGAAAGCTGGGTCTCMGGCGGCGGGGCTGGCC
RipZ	*RSp1031*	pDONR207	pACC82	Oacc23<br>Oacc24	GGAGATAGAACCATGCCGCGTTTTTCAAACTG<br>CAAGAAAGCTGGGTCTCMTCGGCGCATCGACGGCG
RipAM	*RSc3272*	pDONR207	pACC93	Oacc11<br>Oacc12	GGAGATAGAACCATGAAACGAATCGACGTCCAC<br>CAAGAAAGCTGGGTCTCMGGCCGGCCGCGTCGTTG
RipH2	*RSp0215*	pDONR207	pACC98	Oacc13<br>Oacc14	GGAGATAGAACCATGCTTGGCGGAAACATCACGGA<br>CAAGAAAGCTGGGTCTCMATGCGCCCCCGGCGCGC
RipAK	*RSc2359*	pDONR207	pACC113	Oacc9<br>Oacc10	GGAGATAGAACCATGCGCCCTACCGCCCC<br>CAAGAAAGCTGGGTCTCMCAGGTGCGCGATGGCTC
RipAN	*RSp0845*	pDONR207	pACC221	Oacc21<br>Oacc22	GGAGATAGAACCATGCGCCCCCTCTCTTTTC<br>CAAGAAAGCTGGGTCTCMGGCTTCCGGGGCGCCG
RipAW	*RSp1475*	pDONR207	pACC271	Oacc29<br>Oacc30	GGAGATAGAACCATGGTTTTCCTTGTCCGGAGC<br>CAAGAAAGCTGGGTCTCMTCCGCCGCGCGCGGCGAC
RipQ	*RSp1277*	pDONR207	pACC276	Olb69<br>Olb70	GGAGATAGAACCATGCTGCGAACCTCCCTTGAC<br>CAAGAAAGCTGGGTCTCMCACCATGTCGATGGCCG
RipAY	*RSp1022*	pDONR207	pACC385	Olb79<br>Olb80	GGAGATAGAACCATGGAAAGAATCTCGACAA<br>CAAGAAAGCTGGGTCTCMGTCGGGTTTGGGC
RipH1	*RSc1386*	pDONR207	pACC386	Oacc3<br>Oacc4	GGAGATAGAACCATGGCAGGAGGACGAGTCG<br>CAAGAAAGCTGGGTCTCMTCCGGCCGGGCGCACGC
RipR	*RSp1281*	pDONR207	pACC426	Oacc57<br>Oacc58	GGAGATAGAACCATGAACATAAGAAAATACTTAC<br>CAAGAAAGCTGGGTCTCMTTCCAGCGACTTGCTAC

RipW	*RSc2775*	pDONR207	pACC429	Oacc61<br>Oacc62	GGAGATAGAACCATGTCCATCCAGATTGATCGC<br>CAAGAAAGCTGGGTCTCMGCCCGAGTAGGCCTTGTAG
RipTAL	*Rsc1815*	pENTR/D-TOPO	pACC532	Oacc177<br>Oacc178	CACCATGAGAATAGGCAAATCAAGCGGTTGGTTGAAC<br>TCACGTTTCCAATATTTGCAGAAGCCAGTCG
RipAG	*RSc0824*	pDONR207	pACC542	Oacc1<br>Oacc2	GGAGATAGAACCATGGGATGCGTTAATGTC<br>CAAGAAAGCTGGGTCTCMGTCAGAACGGCCGATC
RipV1	*RSc1349*	pDONR207	pACC546	Opr53<br>Opr52	GGAGATAGAACCATGCCAACCCGCGTTCCGTCCCC<br>CAAGAAAGCTGGGTCTCMGCGGCTGCCTTGCGAA
RipH3	*Rsp0160*	pDONR207	pACC588	Olb53<br>Olb54	GGAGATAGAACCATGTTGAAAGGACGTATCGACG<br>CAAGAAAGCTGGGTCTCMCCCGGAAGCCGGCAGCTTGA
RipAJ	*RSc2101*	pDONR207	pACC652	Oacc5<br>Oacc6	GGAGATAGAACCATGCGCCGGACGGCGACTTC<br>CAAGAAAGCTGGGTCTCMCGCTGCGCGGGGCTCGC
RipAD	*Rsp1601*	pENTR/SD/D-TOPO	pACC648	Oacc217<br>Oacc34	GGAGATAGAACCATGTTGAAAATAGGCCGTATC<br>CAAGAAAGCTGGGTCTCMCCAGTCGAGCTGCGGATC
HpaB$_{Xcv\ 85\text{-}10}$	*XCV0416*	pENTR/SD/D-TOPO	pFL47	Ofl82<br>Ofl83	CACCATGAGCAGCGCGCGATTC<br>TCAGGCGCGTAACCACAG
HpaD	*Rsp0848*	pDONR207	pLBy1	Olb17<br>Olb18	GGAGATAGAACCATGCCCTCCGCCGAAACCG<br>CAAGAAAGCTGGGTCTCMATGCGCCTCCCACT
RipI	*RSc0041*	pDONR207	pLBy3	Olb9<br>Olb10	GGAGATAGAACCATGCCTCTTACCAAGATCAATCCTTCGG<br>CAAGAAAGCTGGGTCTCMCTCGGTCGTCCG
RipU	*RSp1212*	pDONR207	pLby7	Olb83<br>Olb84	GGAGATAGAACCATGTCACGCATCTTCAGGTC<br>CAAGAAAGCTGGGTCTCMGCGCGCCTCGTGCG
RipAV	*RSp0732*	pDONR207	pLBy13	Olb77<br>Olb78	GGAGATAGAACCATGATCCCGAAAACACTC<br>CAAGAAAGCTGGGTCTCMCCCCACCGCCTC
RipB	*Rsc0245*	pENTR/SD/D-TOPO	pSC192	RipB-Fwd<br>RipB-Rev	CACCATGAAGGCCGTCACCCGA<br>TCAGCGCGTACCCGGCGT
RipP1 flanked by BsaI sites	*Rsc0826*	pGEM-T	pFL92	Ofl162<br>Ofl163	TTTGGTCTCTTATGAAAAGACTATTCAGAGCATTGG<br>TTTGGTCTCTGATCCGACTCCAGGGCATGTCGA

a

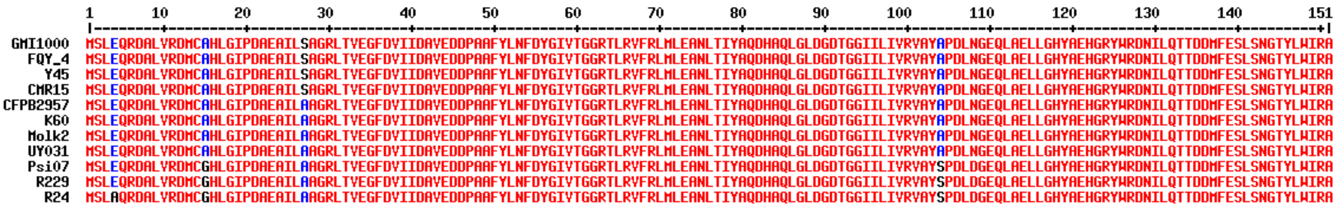

b

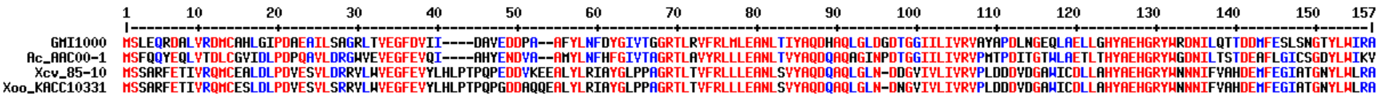

Supplementary Figure S1. (a) Amino acid sequence alignment of HpaB from *R. solanacearum* GMI1000 and its homologs in 10 other wild-type sequenced strains belonging to all four phylotypes. Sequences were aligned using multalin 5.4.1 (http://multalin.toulouse.inra.fr/multalin/). Conserved residues are colored in red. (b) Amino acid sequence alignment of HpaB from *R. solanacearum* GMI1000 and its homologs in *Acidovorax citruli* AAC00-1, *Xanthomonas campestris* pv. *vesicatoria* 85-10 and *Xanthomonas oryzae* pv. *oryzae* KACC10331. Sequences were aligned using multalin 5.4.1 (http://multalin.toulouse.inra.fr/multalin/). Conserved residues are colored in red.

a

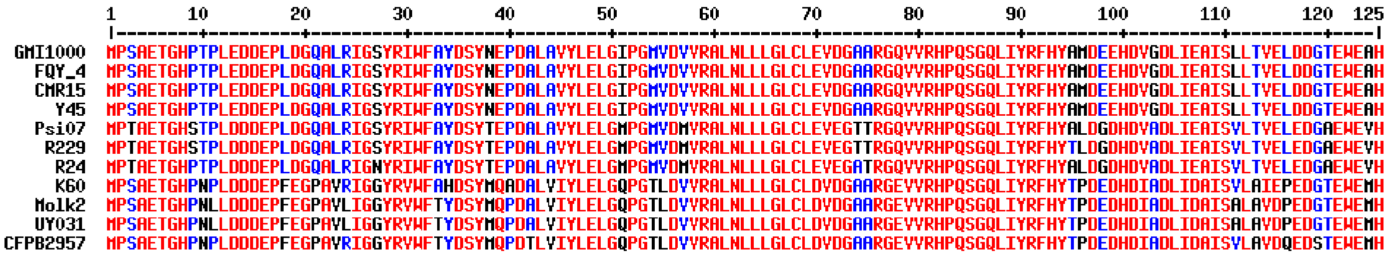

b

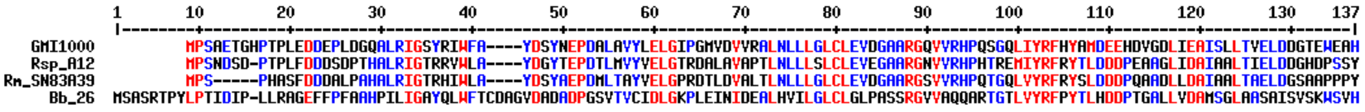

Supplementary Figure S2. (a) Amino acid sequence alignment of HpaD from *R. solanacearum* GMI1000 and its homologs in 10 other wild-type sequenced strains belonging to all four phylotypes. Sequences were aligned using multalin 5.4.1 (http://multalin.toulouse.inra.fr/multalin/). Conserved residues are colored in red. (b) Amino acid sequence alignment of HpaD from *R. solanacearum* GMI1000 and its homologs in *Ralstonia sp* A12, *Ralstonia mannitolilytica* SN83A39 and *Burkholderiaceae bacterium* 26. Sequences were aligned using multalin 5.4.1 (http://multalin.toulouse.inra.fr/multalin/). Conserved residues are colored in red.

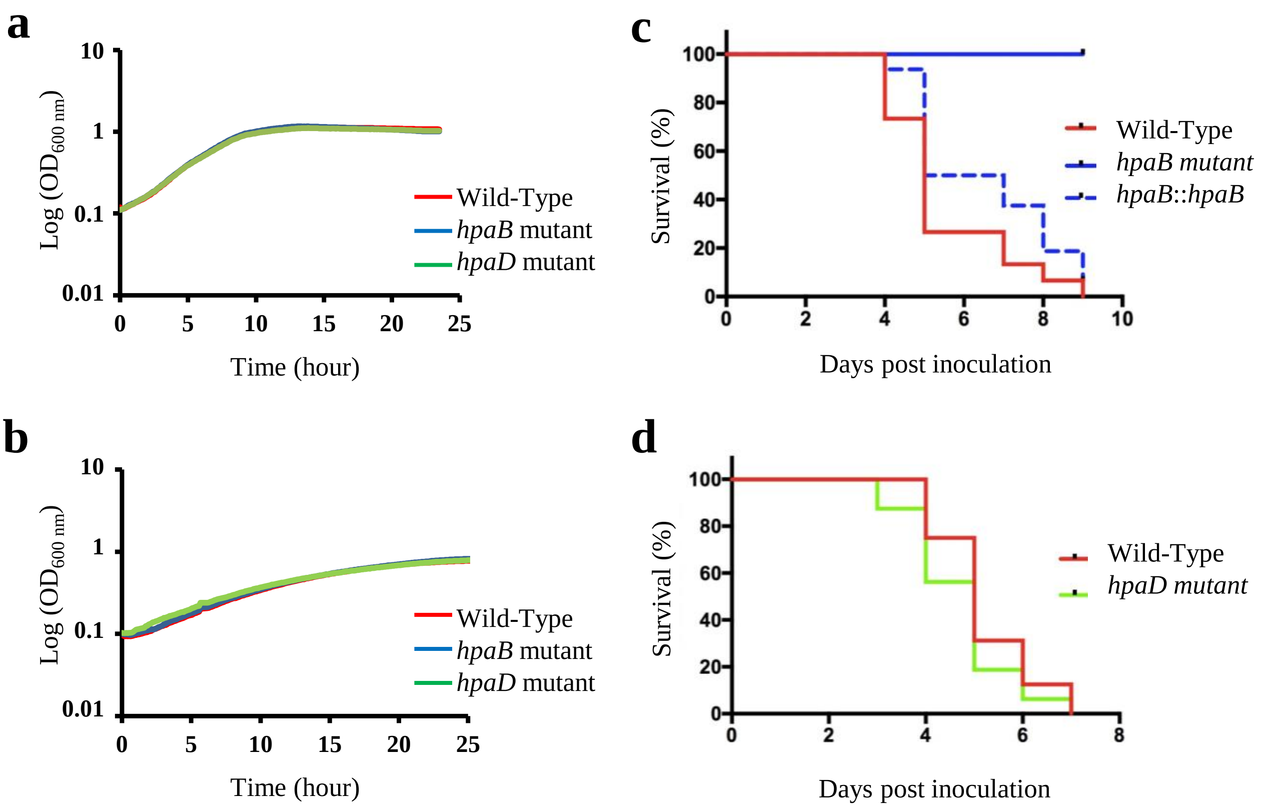

Supplementary Figure S3. HpaB is strictly required for *R. solanacearum* pathogenicity on tomato, not HpaD. *In vitro* growth in complete (a) or minimal medium (b), of the wild-type strain (red), and of the *hpaB* (blue) and *hpaD* (green) mutants. *In vitro* growth was investigated by monitoring OD_{600} during 25 hours starting at 0.1 as described in material and methods. These experiments have been repeated twice. (c) Kaplan-Meier survival analysis of 16 tomato inoculated plants. Gehan-Breslow-Wilcoxon test indicates that the wild-type strain curve (red) is significantly different from the *hpaB* mutant curve (blue) (p value < 0.001), contrary to the complemented *hpaB* curve (dotted blue) (p value $= 0.0678$). (d) Kaplan-Meier survival analysis of 16 tomato inoculated plants. Gehan-Breslow-Wilcoxon test indicates that the wild-type strain curve (red) is not significantly different from the *hpaD* curve (green) (p value $= 0.2689$). These experiments have been repeated three times.

Supplementary references

1. Salanoubat, M. *et al.* Genome sequence of the plant pathogen *Ralstonia solanacearum*. *Nature* **415,** 497–502 (2002).
2. Cunnac, S., Occhialini, A., Barberis, P., Boucher, C. & Genin, S. Inventory and functional analysis of the large Hrp regulon in *Ralstonia solanacearum*: identification of novel effector proteins translocated to plant host cells through the type III secretion system. *Mol. Microbiol.* **53,** 115–128 (2004).
3. Lonjon, F. *et al.* comparative secretome analysis of *Ralstonia solanacearum* type 3 secretion-associated mutants reveals a fine control of effector delivery, essential for bacterial pathogenicity. *Mol. Cell. Proteomics* **15,** 598–613 (2016).
4. Kousik, C. S. & Ritchie, D. F. Response of Bell Pepper Cultivars to Bacterial Spot Pathogen Races that Individually Overcome Major Resistance Genes. *Plant Dis.* **82,** 181–186 (1998).
5. Wengelnik, K., Rossier, O. & Bonas, U. Mutations in the regulatory gene *hrpG* of *Xanthomonas campestris* pv. *vesicatoria* result in constitutive expression of all *hrp* genes. *J. Bacteriol.* **181,** 6828–6831 (1999).
6. Büttner, D., Gürlebeck, D., Noël, L. D. & Bonas, U. HpaB from *Xanthomonas campestris* pv. *vesicatoria* acts as an exit control protein in type III-dependent protein secretion. *Mol. Microbiol.* **54,** 755–768 (2004).
7. Scheibner, F., Schulz, S., Hausner, J., Marillonnet, S. & Büttner, D. Type III-Dependent Translocation of HrpB2 by a Nonpathogenic *hpaABC* Mutant of the Plant-Pathogenic Bacterium *Xanthomonas campestris* pv. *vesicatoria*. *Appl. Environ. Microbiol.* **82,** 3331–3347 (2016).

8. Lohou, D. *et al.* HpaP modulates type III effector secretion in *Ralstonia solanacearum* and harbours a substrate specificity switch domain essential for virulence. *Mol. Plant Pathol.* **15**:601-614 (2014).

9. Poueymiro, M. *et al.* Two type III secretion system effectors from *Ralstonia solanacearum* GMI1000 determine host-range specificity on tobacco. *Mol. Plant-Microbe Interact.* **22,** 538–550 (2009).

10. Solé, M. *et al.* The awr gene family encodes a novel class of *Ralstonia solanacearum* type III effectors displaying virulence and avirulence activities. *Mol. Plant-Microbe Interact.* **25,** 941–953 (2012).

11. Remigi, P., Anisimova, M., Guidot, A., Genin, S. & Peeters, N. Functional diversification of the GALA type III effector family contributes to *Ralstonia solanacearum* adaptation on different plant hosts. *New Phytol.* **192,** 976–987 (2011).

12. Tasset, C. *et al.* Autoacetylation of the *Ralstonia solanacearum* effector PopP2 targets a lysine residue essential for RRS1-R-mediated immunity in Arabidopsis. *PLoS Pathog.* **6,** e1001202 (2010).

13. Guéneron, M., Timmers, A. C., Boucher, C. & Arlat, M. Two novel proteins, PopB, which has functional nuclear localization signals, and PopC, which has a large leucine-rich repeat domain, are secreted through the *hrp*-secretion apparatus of *Ralstonia solanacearum*. *Mol. Microbiol.* **36,** 261–277 (2000).
